# Recurring Themes

**DOI:** 10.1128/mSphere.00633-20

**Published:** 2020-07-08

**Authors:** Michael J. Imperiale

**Affiliations:** a Department of Microbiology and Immunology, University of Michigan, Ann Arbor, Michigan, USA

## EDITORIAL

At the beginning of December 2019, Benhur Lee from our Board of Editors wrote me to ask whether *mSphere* would be interested in publishing a summary of a conference being held at that time in Singapore to commemorate the 20th anniversary of the first Nipah virus outbreak. Having attended a similar conference on Ebola and other emerging infectious diseases a couple of years earlier, I knew that the topics of discussion and the information presented at such a meeting are of interest and importance to the microbial sciences community. I therefore told Benhur that we were absolutely interested: the report from the Nipah@20 conference is published with this editorial (1).

Little did he or I know in early December that another outbreak was simmering at that exact time. Severe acute respiratory syndrome coronavirus 2 (SARS-CoV-2) was beginning its journey from Wuhan, China to the rest of the world, and as of the time I am writing this, well over 10 million cases and half a million deaths have been reported. As the authors of the Nipah@20 conference summary note, the similarities in terms of what the world needs to respond to such emerging diseases are many. What is distressing is that the stories are all too familiar: lack of commitment and resources to fund research, surveillance, and development of therapeutics and vaccines.

We have heard these appeals in the context of viral diseases over and over and over again. HIV/AIDS. Ebola. Nipah. Avian influenza. SARS. COVID-19. When is the world going to realize that we must be better prepared for events that are occurring all too regularly? I strongly encourage you, the scientific community, to advocate vigorously for increased attention to, and funding for, these constantly emerging threats.
